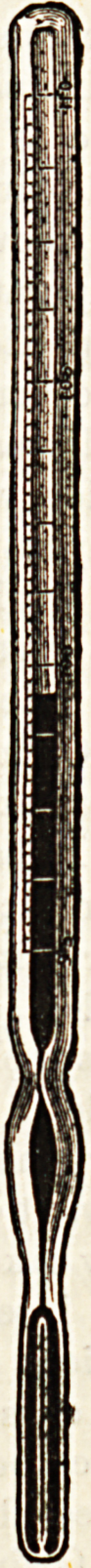# New Drugs, Appliances, and Things Medical

**Published:** 1890-07-05

**Authors:** 


### NEW DRUGS, APPLIANCES, AND THINGS MEDICAL.

[All preparations, appliances, novelties, etc., of which a notice is desired, should be sent to The Editor, The Lodge, Porchester Square, W.]

### THE "RAPID" CLINICAL THERMOMETER.

We have had sent to us a clinical thermometer which embodies a very important improvement. The thermometer at the bedside is, perhaps, the most valuable of all modern instruments of precision. It enables us to differentiate with the utmost certainty between acute cases and those that are not acute. It should be used in practically every new case that comes before the physician. That it is not so used is, no doubt, largely due to the fact that with the ordinary thermometer so much time is required for a proper measurement of the temperature. The thermometer sent to us is patented by Mr. James J. Hicks, of Hatton Garden, and has been invented for taking the temperature of the body in the least possible time. When we state that a perfectly accurate registry of the temperature is indicated in less than a third of a minute, or, to be precise, in 25 seconds, the busy practitioner at once understands what an important advance has been made. The bulb of the instruments consists of two tubes running parallel to each other, but united throughout by a piece of solid glass. By these means the extreme sensitiveness required for rapid registration is secured, and at the same time the bulb is said to be made fully six times stronger than that of the ordinary thermometer. We give a sketch of the instrument, which shows its size and outline. Attention is especially directed to the fact that, notwithstanding the duplex bulb, the thermometer requires a very small case, and will occupy no more room in the pocket than one of the ordinary instruments now in use. No dirt or matter of any kind can pass between the bulbs, which are, therefore, easily kept clean. The advantages claimed are sensitiveness, strength, and cleanliness, and the first of these is undoubtedly of great importance, when the two latter are not sacrificed for it. Messrs. Maw, Son, and Thompson, of Aldersgate Street, have, we understand, taken a large consignment, and they are prepared to supply the profession at once. The price is about the same as for other thermometers.

## Figures and Tables

**Figure f1:**